# Partial-gland Cryoablation Outcomes for Localized Prostate Cancer in Patients with Magnetic Resonance Imaging (MRI)-visible and MRI-invisible Lesions

**DOI:** 10.1016/j.euros.2023.04.017

**Published:** 2023-05-19

**Authors:** Alec Zhu, Sofia Gereta, Tenny R. Zhang, Judith Stangl-Kremser, Richard M. Mora, Daniel J.A. Margolis, Jim C. Hu

**Affiliations:** a Department of Urology, NewYork-Presbyterian Hospital/Weill Cornell Medical Center, New York, NY, USA; b Dell Medical School, University of Texas-Austin Austin, TX, USA

**Keywords:** Prostatic neoplasms, Cryosurgery, Treatment failure

## Abstract

**Background:**

Expert consensus recommends treatment of magnetic resonance imaging (MRI)-visible prostate cancer (PCa). Outcomes of partial-gland ablation (PGA) for MRI-invisible PCa remain unknown.

**Objective:**

To compare recurrence-free survival, adverse events, and health-related quality of life (HRQoL) outcomes following cryoablation of MRI-visible vs invisible PCa.

**Design, setting, and participants:**

We analyzed data for 75 men who underwent cryoablation therapy between January 2017 and January 2022. PCa identified on MRI-targeted and/or adjacent systematic biopsy cores was defined as MRI-visible, whereas PCa identified on systematic biopsy beyond the targeted zone was defined as MRI-invisible.

**Outcome measurements and statistical analysis:**

The primary outcome was recurrence at 12 mo after PGA, defined as the presence of clinically significant PCa (grade group [GG] ≥2) on surveillance biopsy. Adverse events were captured using the Clavien-Dindo classification and HRQoL was captured using the Expanded Prostate Cancer Index-Clinical Practice (EPIC-CP) tool.

**Results and limitations:**

Of the 58 men treated for MRI-visible and 17 treated for MRI-invisible lesions, 51 (88%) and 16 (94%), respectively, had at least one surveillance biopsy performed. There were no statistically significant differences in age, race, body mass index, biopsy GG, prostate-specific antigen, prostate volume, or treatment extent between the MRI-visible and MRI-invisible groups. Median follow-up was 44 mo (interquartile range 17–54) and did not significantly differ between the groups. The recurrence rate at 12 mo did not significantly differ between the groups (MRI-visible 39%, MRI-invisible 19%; *p* = 0.2), and log-rank survival analysis demonstrated no significant difference in recurrence-free survival (*p* = 0.15). Adverse event rates did not significantly differ (MRI-visible 29%, MRI-invisible 53%; *p* = 0.092); no man in the MRI-visible group had a Clavien-Dindo grade ≥III complication, while one subject in the MRI-invisible group had a Clavien-Dindo grade III complication. Median EPIC-CP urinary and sexual function scores were similar for the two groups at baseline and at 12 mo after PGA. Study limitations include the retrospective design and small sample size.

**Conclusions:**

We observed similar cancer control, adverse event, and HRQoL outcomes for MRI-visible versus MRI-invisible PCa in the first comparison of partial-gland cryoablation. Longer follow-up and external validation of our findings are needed to inform patient selection for PGA for MRI-invisible PCa.

**Patient summary:**

Patients with prostate cancer lesions that are not visible on magnetic resonance imaging (MRI) scans who undergo partial gland ablation may have similar treatment outcomes compared to patients with cancer lesions that are visible on MRI.

## Take Home Message

Outcomes after partial gland ablation of magnetic resonance imaging (MRI)-invisible prostate cancer are unknown. We demonstrated that men with MRI-visible and MRI-invisible lesions have similar oncological and functional outcomes and complication rates after cryoablation.

## 1 Introduction

Prostate cancer (PCa) is the most commonly diagnosed solid tumor among US men, with estimated incidence of 268 490 and mortality of 34 500 in 2022 [1]. For healthy men with clinically significant PCa (grade group [GG] ≥2), professional guidelines recommend whole-gland definitive therapy with either radical prostatectomy (RP) or radiation therapy (RT) [2], [3]. However, these treatments have negative effects on health-related quality of life (HRQoL) in terms of urinary and sexual function [4]. New treatment options such as partial gland ablation (PGA) have emerged that may attenuate the negative sequelae of whole-gland treatment while achieving cancer control [5]. American Urological Association (AUA) guidelines currently state that PGA may be considered for intermediate-risk PCa, but there is a lack of high-quality evidence comparing ablation to whole-gland treatments [3]. Nevertheless, interest in PGA for PCa has surged in the USA because of an increase in the use of image guidance for targeted prostate biopsy and recent Food and Drug Administration (FDA) approval of high-intensity focused ultrasound (HIFU) for prostate tissue ablation [6], [7]. Various modalities, including HIFU, cryoablation, laser ablation, irreversible electroporation, and magnetic resonance-guided focused ultrasound surgery (MRgFUS), are currently used for PGA.

Contemporary expert consensus on PGA recommends treatment of clinically significant PCa (GG ≥2) diagnosed via targeted biopsy of MRI-visible lesions [8] on the basis of current evidence on PGA for MRI-visible lesions [9], [10], [11], [12], [13], [14], [15]. While multiparametric MRI improves detection of GG ≥2 PCa, approximately 4–6% of GG ≥2 PCa cases are MRI-invisible and detected via systematic biopsy alone [16], [17]. Treatment outcomes for MRI-invisible PCa have not been reported. Therefore, the primary objective of our study was to compare cancer control outcomes of PGA for treatment of MRI-visible versus MRI-invisible lesions. Secondary outcomes were adverse events and HRQoL. We hypothesized that treatment outcomes after cryoablation for men with MRI-visible and MRI-invisible prostate cancer are similar.

## 2 Patients and methods

### 2.1 Study population

We assessed men treated with partial-gland cryoablation at New York-Presbyterian/Weill Cornell Medical Center from January 2017 to January 2022 (NCT #03492424) [18]. Inclusion criteria were men aged >18 yr diagnosed with clinically significant PCa who underwent nonsalvage treatment. All available prostate MRI scans, performed internally or at an external institution, were reviewed by an experienced genitourinary radiologist using Prostate Imaging-Reporting and Data System (PI-RADS) v2, and three-dimensional reconstruction was performed before targeted biopsy and PGA. Men with PCa on targeted or systematic biopsy adjacent to the MRI-targeted region were categorized as having MRI-visible PCa. Men who underwent targeted biopsy with PCa diagnosed on systematic biopsy outside of the targeted zone (cancer was on the opposite side or not adjacent to the targeted lesion) were categorized as having MRI-invisible PCa. All prostate biopsies and cryoablation procedures were performed by a single surgeon (J.C.H.). Targeted fusion biopsy was performed along with 12-core systematic biopsy, consistent with expert consensus recommendations [8]. Men were counseled that there was a lack of data on long-term cancer control outcomes and comparative studies evaluating PGA and whole-gland therapy. They were also counseled regarding conventional treatment options, including active surveillance (AS), RP, and RT. Men with high-risk PCa (GG 4–5) who underwent PGA had been recommended definitive whole-gland therapy, but opted to undergo PGA instead. All men underwent two cryotherapy treatment cycles. The majority of subjects (96%) underwent focal treatment and were treated with a margin of 1.5 cm beyond the lesion border [19], [20]. The study received institutional review board approval (#1702017952).

**Fig. 1:**
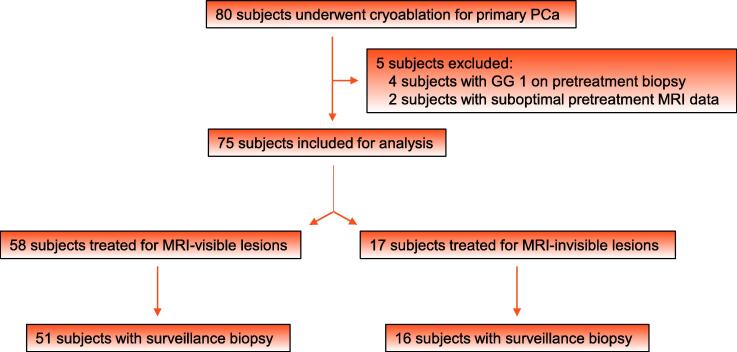
Study flow diagram. PCa = prostate cancer; GG = grade group; MRI = magnetic resonance imaging.

Out of 80 men treated for PGA between January 2017 and January 2022, four were excluded for treatment of GG 1 cancer and two had suboptimal pretreatment MRI scans (Fig. 1). Out of 58 patients with MRI-visible lesions and 17 with MRI-invisible lesions, 51 (87.9%) and 16 (94.1%), respectively, had surveillance biopsy data available.

For our primary outcome of cancer control, we restricted the analysis to men with at least one post-PGA prostate biopsy (*n* = 67). All men were counseled to undergo MRI-targeted biopsy within 6–12 mo of PGA. If initial prostate biopsy was negative for PCa, a second biopsy was performed 24 mo after PGA. Beyond these two initial post-PGA biopsies, additional biopsies were performed based on a suspicious rise in prostate-specific antigen (PSA). Men received serial PSA testing every 6 mo for the first 2 yr and then annually. Recurrence after PGA was defined as the presence of GG ≥2 PCa on surveillance biopsy. In-field and out-of-field recurrences were defined by the presence of PCa within or outside of the PGA ablation zone.

Adverse events within 30 d of cryoablation treatment were captured using the Clavien-Dindo classification. Urinary and sexual function was assessed at pretreatment and post-PGA time points of 3 and 12 mo using the Expanded Prostate Cancer Index for Clinical Practice (EPIC-CP). Urinary continence was defined as answers “0” or “1” to item 3 on EPIC-CP, which reflects use of 0–1 pads/d. Potency was defined as an answer of “0” to item 8, which indicates erections firm enough for intercourse with or without the use of medications.

### 2.2 Statistical analysis

Results for continuous variables were reported as the median with interquartile range (IQR). Continuous variables were compared using the nonparametric Wilcoxon rank-sum test. Categorical variables were compared using Fisher’s exact test. Kaplan-Meier recurrence-free survival curves were generated, and a log-rank test was performed to evaluate for differences in survival between the MRI-visible PCa and MRI-invisible PCa cohorts. Analyses were performed using R v4.2.1 and SAS v9.4. All statistical tests were two-sided, and statistical significance was defined as *p* < 0.05.

## 3 Results

There were no significant differences in demographic characteristics (Table 1) between the MRI-visible PCa (*n* = 51) and MRI-invisible PCa (*n* = 16) groups. However, men with MRI-visible cancer had a greater maximum cancer core length on biopsy (7.0 vs 4.0 mm; *p* = 0.012). In addition, ten men (67%) in the MRI-invisible group had PI-RADS 4 lesions; however, these lesions were not correlated with clinically significant PCa on targeted biopsy and were not treated with cryoablation. The majority of prostate MRI scans were performed internally (70%), and all were reviewed internally by genitourinary radiologists. There were no significant differences between the two groups with regard to treatment laterality, extent, or location.

**Table 1:** Baseline clinical characteristics of men undergoing partial gland cryoablation

Parameter	Overall (*n* = 67)	MRI-visible (*n* = 51)	MRI-invisible (*n* = 16)	*p* value
Median age, yr (IQR)	71 (63.5–75)	71 (64–76)	66.5 (62.8–73)	0.10
Median BMI, kg/m^2^ (IQR)	26.5 (24.9–29.4)	26.0 (24.7–28.2)	28.7 (26.2–30.7)	0.061
Median PSA, ng/ml (IQR)	6.4 (4.7–8.9)	6.4 (4.5–9.1)	6.0 (5–7.9)	0.8
Median MRI volume, ml (IQR)	41.6 (34.0–56.4)	41.0 (33.4–56.4)	48.0 (37.3–56.8)	0.5
Median total biopsy cores, *n* (IQR)	16 (14–18)	16 (14.2–18)	16 (14–18.2)	0.9
Median systematic cores, *n* (IQR)	12 (12–14)	12 (12–14)	13 (12–13.2)	0.4
Median targeted cores, *n* (IQR)	3 (2–5)	3 (2–5)	3 (1.5–4.3)	0.3
Median positive cores, *n* (IQR)	3 (2–5)	4 (3–5)	3 (2–3)	0.016
Median biopsy cores positive, % (IQR)	21 (15–31)	23 (17–33)	15 (10–22)	0.023
Median MCCL, mm (IQR)	6.0 (3.6–9.0)	7.0 (4.1–10.0)	4.0 (2.4–7.0)	0.012
Gleason pattern 4, % (IQR)				
GG 2	10 (5.0–15)	10 (5.0–15)	5.0 (5.0–18)	0.8
GG 3	60 (48–75)	60 (40–70)	73 (64–81)	0.6
Median follow-up, mo (IQR)	44 (17–54)	44 (20–56)	41.5 (14.2–52)	0.3
Race, *n* (%)				0.3
White	35 (52.2)	28 (55)	7 (44)	
Black	6 (9.0)	4 (7.8)	2 (13)	
Asian/Pacific Islander	11 (16.4)	7 (14)	4 (25)	
Hispanic	1 (1.5)	0 (0)	1 (6.3)	
Other/unknown	14 (21)	12 (24)	2 (13)	
Institution where MRI performed, *n* (%)				0.12
External	20 (30)	18 (35)	2 (13)	
Internal	47 (70)	33 (65)	14 (88)	
Highest pretreatment PI-RADS score, *n* (%)				0.002
2	2 (3.0)	0 (0)	2 (13.3)	
3	10 (15)	7 (14)	3 (20)	
4	36 (55)	26 (51)	10 (67)	
5	18 (27)	18 (35)	0 (0)	
Highest pretreatment biopsy GG, *n* (%)				1
GG 2	47 (70)	35 (69)	12 (75)	
GG 3	14 (21)	11 (22)	3 (19)	
GG 4	5 (7.5)	4 (7.8)	1 (6.3)	
GG 5	1 (1.5)	1 (2.0)	0 (0)	
Treatment laterality, *n* (%)				0.6
Unilateral	63 (94)	16 (100)	47 (92)	
Bilateral	4 (6.0)	0 (0)	4 (7.8)	
Treatment extent, *n* (%)				1
Focal	64 (96)	16 (100)	48 (94)	
Hemigland	3 (4.5)	0 (0)	3 (5.9)	
Treatment location, *n* (%)				0.18
Anterior	1 (10)	7 (14)	0 (0)	
Posterior	60 (90)	44 (86)	16 (100)	

BMI = body mass index; GG = grade group; IQR = interquartile range; MCCL = maximum cancer core length; MRI = magnetic resonance imaging; PI-RADS = Prostate Imaging-Reporting and Data System; PSA = prostate-specific antigen.

Over median follow-up of 44 mo (IQR 17–54), 23 men (34%) had PCa recurrence at 12 mo and 29 (43%) had recurrence at 24 mo (Table 2). PCa recurrence rates at 12 mo were similar in the MRI-visible and MRI-invisible groups (39% vs 19%; *p* = 0.2). In-field (43% vs 19%; *p* = 0.14) and out-of-field (26% vs 19%; *p* = 0.7) recurrence rates did not differ significantly between the two groups (Supplementary Table 1). Furthermore, in-field and out-of-field surveillance biopsy results and median cancer core lengths did not differ significantly between the groups. Of the ten men in the MRI-invisible group with PI-RADS 4 lesions on pretreatment MRI, two experienced out-of-field recurrences; neither of these recurrences corresponded to the original PI-RADS 4 lesion.

**Table 2:** Oncological outcomes after partial-gland cryoablation

Parameter	Overall (*n* = 67)	MRI-visible (*n* = 51)	MRI-invisible (*n* = 16)	*p* value
Median time to recurrence, mo (IQR)	9 (7–16)	8.5 (7–16)	9 (7–15)	0.9
Median time to salvage, mo (IQR)	14.5 (9–23.8)	12 (9–23)	23 (17–25)	0.4
Median time to metastasis, mo (IQR)	33 (32.5–33.5)	33 (32.5–33.5)	NA	NA
Recurrence at 12 mo, *n* (%)	23 (34)	20 (39)	3 (19)	0.2
Recurrence at 24 mo, *n* (%)	29 (43)	24 (47)	5 (31)	0.4
Salvage treatment, *n* (%)	27 (40)	23 (45)	4 (25)	0.2
Metastatic disease, *n* (%)	2 (3.0)	2 (3.9)	0 (0)	1
First biopsy within 12 mo, *n* (%)	58 (87)	44 (86)	14 (88)	1
Number of surveillance biopsies, *n* (%)				0.9
1 biopsy	45 (67)	33 (65)	12 (75)	
2 biopsies	18 (27)	14 (28)	4 (25)	
3 biopsies	3 (4.5)	3 (5.9)	0 (0)	
4 biopsies	1 (1.5)	1 (2.0)	0 (0)	
Overall post-PGA biopsy, *n* (%)				0.5
Benign	18 (27)	13 (26)	5 (31)	
GG 1	14 (21)	8 (16)	6 (38)	
GG 2	21 (31)	17 (33)	4 (25)	
GG 3	8 (12)	7 (14)	1 (6.3)	
GG 4	4 (6.0)	4 (7.8)	0 (0)	
GG 5	2 (3.0)	2 (3.9)	0 (0)	
First post-PGA biopsy, *n* (%)				0.9
Benign	25 (37)	19 (37)	6 (38)	
GG 1	14 (21)	9 (18)	5 (31)	
GG 2	20 (30)	16 (31)	4 (25)	
GG 3	6 (9.0)	5 (9.8)	1 (6.3)	
GG 4	1 (1.5)	1 (2.0)	0 (0)	
GG 5	1 (1.5)	1 (2.0)	0 (0)	
Second post-PGA biopsy, *n* (%)				0.9
Benign	8 (38)	6 (35)	2 (50)	
GG 1	6 (29)	4 (24)	2 (50)	
GG 2	2 (9.5)	2 (12)	0 (0)	
GG 3	1 (4.8)	1 (5.9)	0 (0)	
GG 4	3 (14)	3 (18)	0 (0)	
GG 5	1 (4.8)	1 (5.9)	0 (0)	
Salvage treatment, *n* (%)				0.19
Partial gland ablation	11 (41)	11 (48)	0 (0)	
Radical prostatectomy	9 (33)	7 (30)	2 (50)	
Radiotherapy without ADT	3 (11)	2 (8.7)	1 (25)	
Radiotherapy with ADT	4 (15)	3 (13)	1 (25)	

ADT = androgen deprivation therapy; GG = grade group; IQR = interquartile range; MRI = magnetic resonance imaging; NA = not applicable; PGA = partial-gland ablation.

**Fig. 2:**
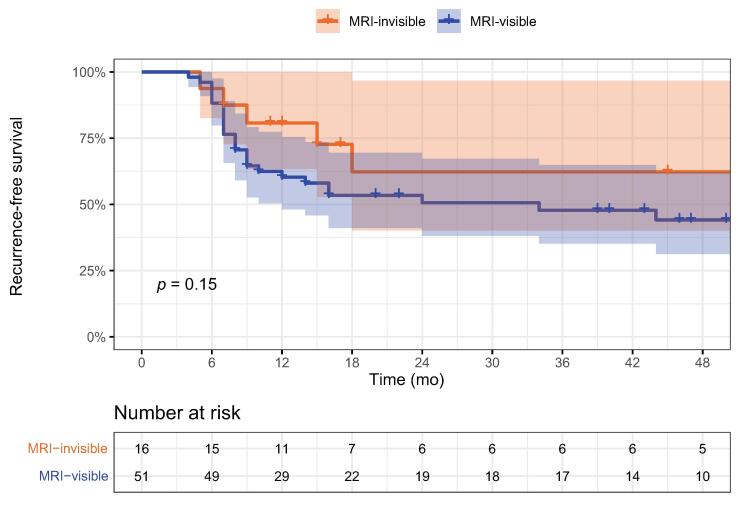
Kaplan-Meier curves for recurrence-free survival after cryoablation of MRI-visible and MRI-invisible lesions (log-rank test, p=0.15). Blue and yellow shading demonstrates 95% confidence intervals, and tick marks represent subject censorship. MRI = magnetic resonance imaging.

The first surveillance biopsy within 12 mo of treatment was performed in 86% of the MRI-visible cases and 88% of the MRI-invisible cases. PCa GG found on first and second surveillance biopsies did not differ significantly following PGA of MRI-visible vs MRI-invisible lesions (Table 2). A log-rank survival analysis demonstrated no significant differences between the two groups (Fig. 2). Recurrence-free survival rates at various times are listed in Supplementary Table 2.

Of the 35 men with recurrence after PGA overall, eight pursued AS while the rest opted for salvage therapy. There were no significant differences in the choice of salvage therapies between the two groups (Table 2). Of the six men with pretreatment GG 4–5 PCa, two developed recurrence within 12 mo and one developed recurrence at 47 mo.

Analysis was also performed to compare the group with at least one surveillance biopsy and the group with no surveillance biopsy. There were no significant differences in age, race, PSA, pretreatment biopsy GG, or other baseline characteristics between these subgroups (Supplementary Table 3). The only difference noted was that men with surveillance biopsy had a higher rate of focal treatment than men without follow-up biopsy (96% vs 63%; *p* = 0.014).

Additional analysis was performed for the 11 men who had salvage cryoablation after initial recurrence. All subjects initially had MRI-visible lesions, and 73% had an initial surveillance biopsy within 12 mo after salvage treatment. The 12-mo recurrence rate after salvage therapy was 44.4%, and one patient developed metastatic disease 32 mo after treatment (Supplementary Table 4).

### 3.1 Functional outcomes

All men with available EPIC-CP data (*n* = 45) were continent (0–1 pads/d) at baseline and at 12 mo after treatment. Of the 36 men (84%) who were potent at baseline, 16 (76%) remained potent at 12 mo after cryoablation. There were no significant differences between the MRI-visible and MRI-invisible groups with regard to baseline (81% vs 92%; *p* = 0.7) and 12-mo (77% vs 75%; *p* = 1) potency rates. In addition, there were no significant differences in median urinary function or sexual function EPIC-CP scores at baseline or at 3 mo and 12 mo after treatment (Table 3).

**Table 3:** Urinary and sexual function outcomes after partial-gland cryoablation

Parameter	Overall	MRI-visible	MRI-invisible	*p* value
Continent (0–1 pads/d), *n* (%)				
Baseline	44 (100)	32 (100)	12 (100)	NA
12 mo	22 (100)	17 (100)	5 (100)	NA
Potent, *n* (%)				
Baseline	36 (84)	25 (81)	11 (92)	0.7
12 mo	16 (76)	13 (77)	3 (75)	1
Median urinary function score (IQR)				
Baseline	0 (0–1)	0 (0–0.25)	0 (0–1)	0.4
3 mo	0 (0–0)	0 (0–0.5)	0 (0–0)	0.5
12 mo	0 (0–1)	0 (0–1)	0 (0–0.125)	0.5
Median sexual function score (IQR)				
Baseline	1 (0–5)	1 (0–5)	1.5 (0–3.5)	0.7
3 mo	3.5 (1–6)	3 (1.25–7.25)	4.5 (0–6)	1
12 mo	3 (1–6)	1 (1–6)	5 (5–6)	0.3

IQR = interquartile range; MRI = magnetic resonance imaging.

### 3.2 Adverse events

Twenty-six men (35%) experienced adverse events within 30 d of cryoablation. The majority were limited to Clavien-Dindo grade I/II events (96%; Table 4). One man developed postcryotherapy prostatic infection with MRI findings that suggested the presence of an abscess; cystoscopy and transurethral unroofing were performed without demonstration of frank pus (Clavien-Dindo grade IIIB). There were no significant differences in rates of any adverse events (29% vs 53%; *p* = 0.092) or severe adverse events (0% vs 5%; *p* = 0.3) between the MRI-visible and MRI-invisible groups.

**Table 4:** Adverse events by Clavien-Dindo grade within 30 d of partial-gland cryoablation

Parameter	Overall (*n* = 75)	MRI-visible (*n* = 58)	MRI-invisible (*n* = 17)	*p* value
Any adverse event, *n* (%)	26 (35)	16 (29)	10 (53)	0.092
Severe adverse event (grade III–V), *n* (%)	1 (1.3)	0 (0)	1 (5.3)	0.3
Clavien-Dindo grade I, *n* (%)	22 (85)	15 (94)	7 (70)	0.2
Clavien-Dindo grade II, *n* (%)	3 (12)	1 (6.3)	2 (20)	
Clavien-Dindo grade III, *n* (%)	1 (3.8)	0 (0)	1 (10.0)	
Clavien-Dindo grade IV/V, *n* (%)	0 (0)	0 (0)	0 (0)	
Adverse event by type)				
Urinary retention	17 (23)	12 (21)	5 (26)	0.8
Urinary tract infection	2 (2.7)	0 (0)	2 (11)	0.062
Hematuria	12 (16)	6 (11)	6 (32)	0.064
Rectal/perineal pain	6 (8.0)	5 (8.9)	1 (5.3)	1
Epididymo-orchitis	2 (2.7)	1 (1.8)	1 (5.3)	0.4
Prostatic abscess	1 (1.3)	0 (0)	1 (5.3)	0.3

MRI = magnetic resonance imaging.

## 4 Discussion

PGA is an emerging treatment option for localized PCa with the aim of oncological control and preservation of HRQoL. While previous PGA series have evaluated oncological and functional outcomes for MRI-targetable lesions, our series is the first to compare outcomes between MRI-visible and MRI-invisible lesions. In addition, we present biopsy endpoints in accordance with FDA-driven expert consensus for evaluation of PGA as a therapeutic option in light of a significant evidence gap, as defined by AUA and European Association of Urology guidelines [3], [21], [22]. We found overall recurrence rates of 34% at 12 mo at 43% at 24 mo. While MRI-visible cancers had a higher tumor volume according to the maximum cancer core length on biopsy, consistent with prior work demonstrating that MRI detection sensitivity correlates with tumor size [23], there was no significant difference in recurrence rate for MRI-visible versus MRI-invisible lesions at 12 mo (39% vs 19%) or 24 mo (47% vs 31%).

Our results reveal higher recurrence rates after cryoablation in comparison to other series, but different definitions of cancer recurrence have been used. For example, Shah et al [9] found a failure-free survival rate of 90.5% at 3 yr among 122 patients who underwent cryoablation, and the surveillance protocol involved PSA testing, MRI, and “for-cause” biopsies if recurrence was suspected. Baskin et al [24] observed a failure-free survival rate of 96% at 2 yr after cryoablation, with failure defined as transition to radical treatment for biochemical recurrence according to the Phoenix criteria [24].

There is significant variation in PGA cancer control endpoints across studies. Expert consensus advocates for objective oncological endpoints such as PCa recurrence on biopsy rather than the need for salvage RP or RT [8], [22]. Multiple series have defined recurrence as the need for salvage treatment, and few series capture post-PGA surveillance biopsy endpoints [9], [10], [11], [12], [13], [25], [26]. In an analysis of 160 men who underwent hemigland cryoablation, of whom 104 who had follow-up biopsy, Oishi et al [25] found that recurrence—defined as clinically significant PCa on surveillance biopsy—was evident in 15% at 3 yr and 37% at 5 yr. Although our treatment success rate is lower than in this study, the results may reflect differences such as our greater use of PGA rather than a hemigland approach and a greater proportion of subjects (89%) with surveillance biopsy data in our study.

Recent studies evaluating HIFU and MRgFUS used a pathological endpoint based on surveillance biopsy to define oncological efficacy, consistent with FDA-defined outcomes as a pathway towards approval for a PCa indication [12], [13], [14], [15], [26], [27]. Our study examined the same biopsy-defined endpoint through an initial surveillance biopsy 6–12 mo after PGA and, if negative for cancer, a second surveillance biopsy at 24 mo after PGA. Results from a recent multicenter trial in men with MRI-visible intermediate-risk PCa demonstrated a recurrence rate of 40% at 24 mo after MRgFUS [14]. This finding is significant because the trial used more stringent patient selection criteria than in our study, as well as real-time in-bore targeting. While expert consensus is to perform PGA on MRI-visible lesions, it is unclear if PGA may be performed for MRI-invisible lesions. Our study is the first to demonstrate that oncological and functional outcomes after PGA are similar for subjects with MRI-visible versus MRI-invisible lesions.

Our results also demonstrate a minimal change in EPIC-CP scores at 12 mo after cryoablation, which compares favorably to patient-reported outcomes after whole-gland therapy with surgery or radiation [4]. These favorable functional outcomes are similar to those in other cryoablation PGA series [9], [24], [25]. The rate of adverse events was 35%, and the rate of severe adverse events (Clavien-Dindo grade ≥III) was low at 1%, both of which are comparable to previously reported outcomes [9].

Our study must be interpreted in the context of its design. First, our definition of MRI-invisible lesions included PCa found on systematic biopsy that was on the opposite side or not adjacent to the MRI target(s). While independent review of prostate MRI and pathology reports was performed to define these lesions, discrepancies may be possible if performed by multiple independent reviewers. In addition, the proportion of clinically significant PCa detected on systematic biopsy outside of the MRI region of interest can vary according to which pathological endpoints are used. Our definition of MRI-invisible PCa is based on positivity in a systematic biopsy core(s) rather than MRI correlation with whole-mount specimens, whereby a higher proportion of men have MRI-invisible cancer [16], [23], [28]. Second, our primary outcome of interest was overall recurrence rather than in-field recurrence of clinically significant PCa. Although we assessed in-field and out-of-field recurrences after PGA, this classification is challenged by inaccuracies in post-treatment MRI. Cryoablation may alter the prostate size and orientation on both the treated ipsilateral and untreated contralateral sides, thereby affecting the reliability of targeted biopsies in the treated regions [29]. In addition, overall recurrence, rather than in-field recurrence, is more clinically relevant and ultimately guides clinical decision making. Third, our study has limited follow-up with a median time of 44 mo. In our survival analysis comparing MRI-visible and MRI-invisible PCa, a significant number of subjects were censored after the first 12 mo, either because of recurrence or lack of a second surveillance biopsy. Although we report salvage treatment plans and metastases, long-term outcomes such as cancer-specific and overall mortality have not matured. Nevertheless, of the 42 men with at least 2 yr of follow-up, 22 (52%) received at least two surveillance biopsies after treatment, which is consistent with consensus recommendations [8], [22], [30] and less commonly reported than PSA or salvage therapy endpoints for cancer control. Additional limitations include our retrospective study design and the absence of a comparison arm.

## 5 Conclusions

PGA is a safe treatment option for clinically localized PCa, with minimal effects on HRQoL and a favorable adverse event profile. Persistence or recurrence of clinically significant PCa was evident in approximately half of patients treated with partial-gland cryoablation. Although our recurrence-free survival rates are somewhat lower than previously published outcomes, our study used pathological endpoints to define cancer recurrence and is the first to demonstrate similar outcomes for MRI-visible and MRI-invisible lesions. While a central dogma for PGA is that appropriate candidates for treatment are men with MRI-visible lesions, we demonstrated that men with systematic biopsy–positive, MRI-invisible lesions may be treated with similar safety, HRQoL, and cancer control outcomes. Larger sample sizes and prospective trials are needed to validate our findings.

  ***Author contributions***: Jim C. Hu had full access to all the data in the study and takes responsibility for the integrity of the data and the accuracy of the data analysis.

  *Study concept and design*: Zhu, Hu.

*Acquisition of data*: Zhu, Gereta, Stangl-Kremser, Mora, Margolis, Hu.

*Analysis and interpretation of data*: Zhu, Margolis, Hu.

*Drafting of the manuscript*: Zhu, Zhang, Hu.

*Critical revision of the manuscript for important intellectual content*: Zhu, Zhang, Margolis, Hu.

*Statistical analysis*: Zhu.

*Obtaining funding*: Hu.

*Administrative, technical, or material support*: Zhu, Margolis, Hu.

*Supervision*: Hu.

*Other*: None.

  ***Financial disclosures:*** Jim C. Hu certifies that all conflicts of interest, including specific financial interests and relationships and affiliations relevant to the subject matter or materials discussed in the manuscript (eg, employment/affiliation, grants or funding, consultancies, honoraria, stock ownership or options, expert testimony, royalties, or patents filed, received, or pending), are the following: Jim C. Hu is a consultant for Intuitive Surgical and Pfizer. The remaining authors have nothing to disclose.

  ***Funding/Support and role of the sponsor*:** Jim C. Hu has received research support from the Frederick J. and the Theresa Dow Wallace Fund of the New York Community Trust, and salary support from NIH R01 CA241758, NIH R01 CA259173, NIH R01 CA273031, the Prostate Cancer Foundation, PCORI CER-2019C1-15682, and CER-2019C2-17372. These sponsors played no direct role in the study.

  ***Acknowledgments***: The authors would like to acknowledge Spyridon P. Basourakos, MD, Leonardo D. Borregales, MD, and Zorawar Singh, MD, for their contributions to maintaining the partial gland ablation database.

  ***Data sharing statement***: Data are available for researchers on request from the authors.
