# Supplementary material for: Partial-gland Cryoablation Outcomes for Localized Prostate Cancer in Patients with Magnetic Resonance Imaging (MRI)-visible and MRI-invisible Lesions
Source: Eur Urol Open Sci. 2023 May 19;53:38–45. doi: 10.1016/j.euros.2023.04.017 (PMC10334232; doi:10.1016/j.euros.2023.04.017)
Supplement: Supplementary data 1 [file mmc1.docx]

**Supplemental Table 1**. In-field and out-of-field biopsy outcomes after partial gland cryoablation

| **n (%)** | **Overall n=67** | **Treatment of MRI visible lesions n=51** | **Treatment of MRI invisible lesions n=16** | **p-value** |
| --- | --- | --- | --- | --- |
| **Total Recurrences after PGA** | 35 (52) | 30 (59) | 5 (31) | 0.084 |
| **In-field recurrence** | 25 (37) | 22 (43) | 3 (19) | 0.14 |
| **Out-of-field recurrence** | 16 (24) | 13 (26) | 3 (19) | 0.7 |
| **In-field biopsy** |  |  |  | 0.4 |
| Benign | 36 (54) | 26 (51) | 10 (63) |  |
| **GG** |  |  |  |  |
| 1 | 6 (9.0) | 3 (5.9) | 3 (19) |  |
| 2 | 17 (25) | 14 (28) | 3 (19) |  |
| 3 | 6 (9.0) | 6 (12) | 0 (0) |  |
| 4 | 1 (1.5) | 1 (2.0) | 0 (0) |  |
| 5 | 1 (1.5) | 1 (2.0) | 0 (0) |  |
| **Out-of-field biopsy** |  |  |  | 0.7 |
| Benign | 34 (51) | 27 (53) | 7 (44) |  |
| **GG** |  |  |  |  |
| 1 | 17 (25) | 11 (22) | 6 (38) |  |
| 2 | 12 (18) | 10 (20) | 2 (13) |  |
| 3 | 2 (3.0) | 1 (2.0) | 1 (6.3) |  |
| 4 | 1 (1.5) | 1 (2.0) | 0 (0) |  |
| 5 | 1 (1.5) | 1 (2.0) | 0 (0) |  |
|  |  |  |  |  |
| **Median (IQR)** |  |  |  |  |
| In-field biopsy |  |  |  |  |
| Cancer core length, mL | 4 (2-6.5) | 4 (2-6.8) | 3 (1.4-4.6) | 0.4 |
| Out-of-field biopsy |  |  |  |  |
| Cancer core length, mL | 2.5 (1-5) | 2 (1-5) | 3 (1-4) | 0.4 |

**Supplemental Table 2**. Survival analysis for MRI-visible vs. invisible cohorts.

| **MRI-visible** |  |  |  |  |  |
| --- | --- | --- | --- | --- | --- |
| Time (months) | Number at risk, n | Failure event, n | survival (%) | standard error | 95% confidence interval |
| 0 | 51 | 0 | 100 | 0 | 100-100 |
| 6 | 49 | 6 | 88 | 4.5 | 80-98 |
| 12 | 29 | 14 | 60 | 6.9 | 48-76 |
| 18 | 22 | 3 | 53 | 7.2 | 41-70 |
| 24 | 19 | 1 | 51 | 7.3 | 38-67 |
| 30 | 18 | 0 | 51 | 7.3 | 38-67 |
| 36 | 17 | 1 | 48 | 7.4 | 35-65 |
| 42 | 14 | 0 | 48 | 7.4 | 35-65 |
| 48 | 10 | 1 | 44 | 7.7 | 31-62 |
|  |  |  |  |  |  |
| **MRI-invisible** |  |  |  |  |  |
| Time, months | Number at risk, n | Failure event, n | survival (%) | standard error | 95% confidence interval |
| 0 | 16 | 0 | 100 | 0 | 100-100 |
| 6 | 15 | 1 | 94 | 6.1 | 83-100 |
| 12 | 11 | 2 | 81 | 10 | 63-100 |
| 18 | 7 | 2 | 62 | 14 | 40-97 |
| 24 | 6 | 0 | 62 | 14 | 40-97 |
| 30 | 6 | 0 | 62 | 14 | 40-97 |
| 36 | 6 | 0 | 62 | 14 | 40-97 |
| 42 | 6 | 0 | 62 | 14 | 40-97 |
| 48 | 5 | 0 | 62 | 14 | 40-97 |

**Supplemental Table 3**. Baseline clinical characteristics of subjects who had at least one surveillance biopsy vs. subjects with no surveillance biopsies

| **Median (IQR)** | **Surveillance biopsy**  **n=67** | **No surveillance biopsy**  **n=8** | **p-value** |
| --- | --- | --- | --- |
| **Age** | 71 (63.5-75) | 75 (67-77.2) | 0.2 |
| **BMI, kg/m^2^** | 26.5 (24.9-29.4) | 24.4 (23.5-27.6) | 0.19 |
| **PSA, ng/mL** | 6.4 (4.7-8.9) | 8.1 (6.1-11.4) | 0.18 |
| **MRI volume, mL** | 41.6 (34.0-56.4) | 46.8 (34.0-66.0) | 0.7 |
| **Total biopsy cores** | 16 (14-18) | 16 (14.5-17.5) | 0.8 |
| **Total sys cores** | 12 (12-14) | 12 (12-13.5) | 0.9 |
| **Total target cores** | 2 (2-5) | 3 (2-4.5) | 0.8 |
| **Total pos cores** | 3 (2-5) | 3 (2.5-4) | 0.7 |
| **Percent biopsy cores positive** | 21.0 (14.05-31.0) | 18.8 (15.8-23.6) | 0.6 |
| **Follow-up, months** | 44 (17-54) | 20.5 (17.2-31.2) | 0.070 |
|  |  |  |  |
| **n (%)** |  |  |  |
| **Race** |  |  | 0.10 |
| White | 35 (52) | 2 (25) |  |
| Black | 6 (9.0) | 0 (0) |  |
| Asian/Pacific Islander | 11 (16) | 1 (13) |  |
| Hispanic | 1 (1.5) | 1 (13) |  |
| Other/Unknown | 14 (21) | 4 (50) |  |
|  |  |  |  |
| **MRI institution** |  |  | 0.7 |
| External | 20 (30) | 3 (38) |  |
| Internal | 47 (70) | 5 (63) |  |
|  |  |  |  |
| **Pre-treatment highest PI-RADS** |  |  | 0.8 |
| 2 | 2 (3.0) | 0 (0) |  |
| 3 | 10 (15) | 0 (0) |  |
| 4 | 36 (55) | 5 (63) |  |
| 5 | 18 (27) | 3 (38) |  |
|  |  |  |  |
| **Pre-treatment highest biopsy GG** |  |  | 0.8 |
| 2 | 47 (70) | 7 (88) |  |
| 3 | 14 (21) | 1 (13) |  |
| 4 | 5 (7.5) | 0 (0) |  |
| 5 | 1 (1.5) | 0 (0) |  |
| **Treatment laterality** |  |  | 0.4 |
| Unilateral | 63 (94) | 7 (88) |  |
| Bilateral | 4 (6.0) | 1 (13) |  |
| **Treatment extent** |  |  | 0.014 |
| Focal | 64 (96) | 5 (63) |  |
| Hemi-gland | 3 (4.5) | 3 (38) |  |
| **Treatment location** |  |  | 0.2 |
| Anterior | 7 (10) | 2 (25) |  |
| Posterior | 60 (90) | 6 (75) |  |
| **Salvage Treatment** | 27 (40) | 0 (0) | *NA* |
| **Metastatic disease** | 2 (3.0) | 0 (0) | *NA* |

**Supplemental Table 4**. Oncologic outcomes after salvage partial gland cryoablation.

| **n (%)** | **MRI-visible n=11** |
| --- | --- |
| **Initial recurrence location** |  |
| In-field | 6 (55) |
| Out-of-field | 3 (27) |
| Both in-field and out-of-field | 2 (18) |
| **Recurrence at 12 months after salvage** | 4 (44) |
| **Recurrence at 24 months after salvage** | 7 (78) |
| **Total Recurrences after salvage** | 7 (70) |
| **Salvage treatment** | 5 (50) |
| **Metastatic disease** | 1 (9.1) |
| **First biopsy within 12 months** | 8 (73) |
| **No. of surveillance biopsies post-salvage** |  |
| 0 biopsies | 1 (9.1) |
| 1 biopsy | 6 (55) |
| 2 biopsies | 4 (36) |
| **Overall post-salvage biopsy** |  |
| Benign | 1 (10) |
| **GG** |  |
| 1 | 2 (20) |
| 2 | 2 (20) |
| 3 | 3 (30) |
| 4 | 1 (10) |
| 5 | 1 (10) |
| **In-field post-salvage biopsy** |  |
| Benign | 2 (20) |
| **GG** |  |
| 1 | 1 (10) |
| 2 | 3 (30) |
| 3 | 3 (30) |
| 4 | 1 (10) |
| 5 | 0 (0) |
| **Out-of-field post-salvage biopsy** |  |
| Benign | 6 (60) |
| **GG** |  |
| 1 | 2 (20) |
| 2 | 1 (10) |
| 3 | 0 (0) |
| 4 | 0 (0) |
| 5 | 1 (10) |
| **Secondary salvage** **treatment** |  |
| Radical Prostatectomy | 2 (40) |
| Radiotherapy without ADT | 2 (40) |
| Radiotherapy with ADT | 1 (20) |
|  |  |
| **Median (IQR)** |  |
| **Time to recurrence, months** | 12 (8-17) |
| **Time to salvage, months** | 14 (12-17) |
| **Time to metastasis, months** | 32 (32-32) |
|  |  |
| **In-field biopsy** |  |
| Cancer core length, mL | 4 (1.9-6.5) |
| **Out-of-field biopsy** |  |
| Cancer core length, mL | 3.5 (2-5.8) |
